# Characterization of Teicoplanin-Specific T-Cells from Drug Naïve Donors Expressing HLA-A*32:01

**DOI:** 10.1021/acs.chemrestox.1c00425

**Published:** 2022-02-02

**Authors:** Joshua Gardner, Monday Ogese, Catherine J. Betts, Munir Pirmohamed, Dean J. Naisbitt

**Affiliations:** † Department of Molecular and Clinical Pharmacology, University of Liverpool, Sherrington Building, Ashton Street, Liverpool L69 3GE, United Kingdom; ‡ Clinical Pharmacology and Safety Sciences, R&D Biopharmaceuticals, AstraZeneca, Cambridge CB4 0WG, United Kingdom

## Abstract

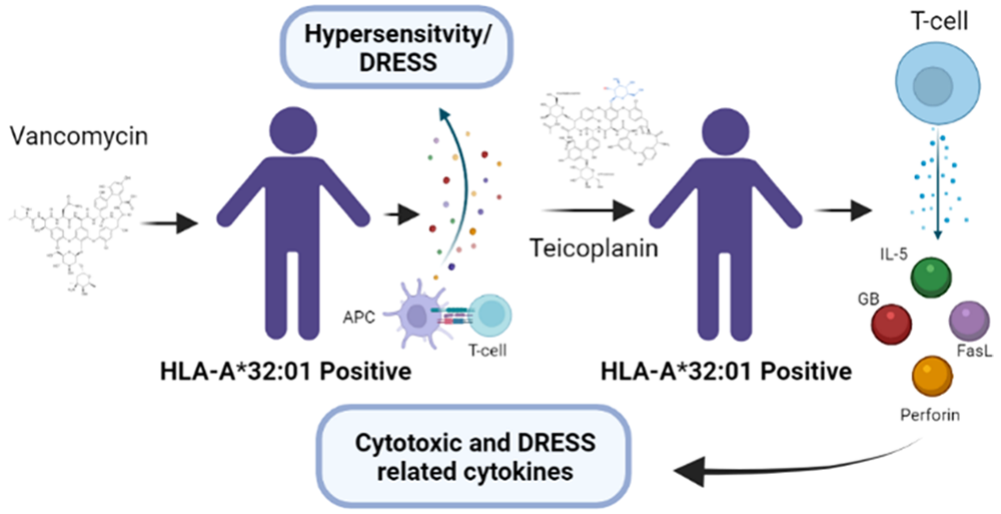

Teicoplanin is a glycopeptide antibiotic deployed to combat Gram-positive bacterial infection and has recently been associated with development of adverse drug reactions, particularly following previous exposure to vancomycin. In this study, we generated teicoplanin-specific monoclonal T-cell populations from healthy volunteers expressing HLA-A*32:01 and defined pathways of T-cell activation and HLA allele restriction. Teicoplanin-responsive T-cells were CD8+, HLA class I-restricted, and cross-reacted with the lipoglycopeptide daptomycin in proliferation and cytokine/cytolytic molecule (granzyme B, Perforin, and FasL) release assays. These data show that teicoplanin activates T-cells, which may play a role in the pathogenesis of teicoplanin-induced adverse events, in HLA-A*32:01 positive donors.

Hypersensitivity to otherwise efficacious antibiotics is an area of concern to patients, clinicians, and researchers in the field of drug development. Prediction of such reactions is often difficult due to the elicitation of adverse events arising outside of a drug’s known pharmacology. Although rare, reactions of this nature have been associated with activation of the adaptive immune system, with T-cells implicated in the pathogenesis of severe cutaneous adverse reaction, including drug-reaction with eosinophilia and systemic symptoms (DRESS).^1^ Glycopeptide antibiotics, such as teicoplanin, have been utilized for over 30 years with strong efficacy demonstrated against Gram-positive bacterial infection, including β-lactam resistant strains such as methicillin-resistant *Staphylococcus aureus* (MRSA) and *Clostridium difficile*.^2^ Teicoplanin is typically administered as a second line treatment option and as an alternative to vancomycin. Despite the incidence of adverse drug reaction (ADR) associated with teicoplanin being substantially lower (13.9% vs 21.9%^3^) compared to vancomycin, the drug still poses a significant risk to patient safety. A recent GWAS has shown an association between vancomycin-induced DRESS and HLA-A*32:01 in European populations.^4^ Case studies have reported clinical cross-reactivity and subsequent teicoplanin-induced DRESS following initial vancomycin hypersensitivity.^5,6^ Preliminary *in vitro* studies using vancomycin-responsive T-cells generated from HLA-A*32:01 positive healthy donor PBMCs have already demonstrated low levels of cross-reactivity with teicoplanin.^7^ Cross-reactivity has been illustrated further in patients presenting with suspected vancomycin or teicoplanin-induced DRESS, with *ex vivo* data suggesting complex patterns of immunogenicity within the context of HLA class II presentation.^8^ The aim of the present study was to investigate the intrinsic immunogenic potential of teicoplanin in terms of evoking T-cell responses in healthy donors (HDs), in addition to further exploring patterns of cross-reactivity to structurally related glycopeptides.

Teicoplanin-specific T-cell clones (TCCs), generated by serial dilution,^9^ were identified in 3 healthy donors positive for HLA-A*32:01 expression (Figure 1). TCCs generated from CD8+ enriched populations proliferated to a greater degree (HD-2, 3; SI > 40) and frequency (HD-1; 118/216 TCC SI > 2) than CD4+ enriched. The presence of drug-reactive T-cells that proliferated in a dose-dependent manner to teicoplanin (data not shown) was restricted to monoclonal populations enriched for CD8+ T-cells, as upon expansion, CD4+ TCCs did not respond to teicoplanin following confirmatory dose–response tests. Drug-responsive clonal populations that exclusively expressed a CD8+ phenotype were expanded via mitogen driven stimulation for further functional analysis.

**Figure 1:**
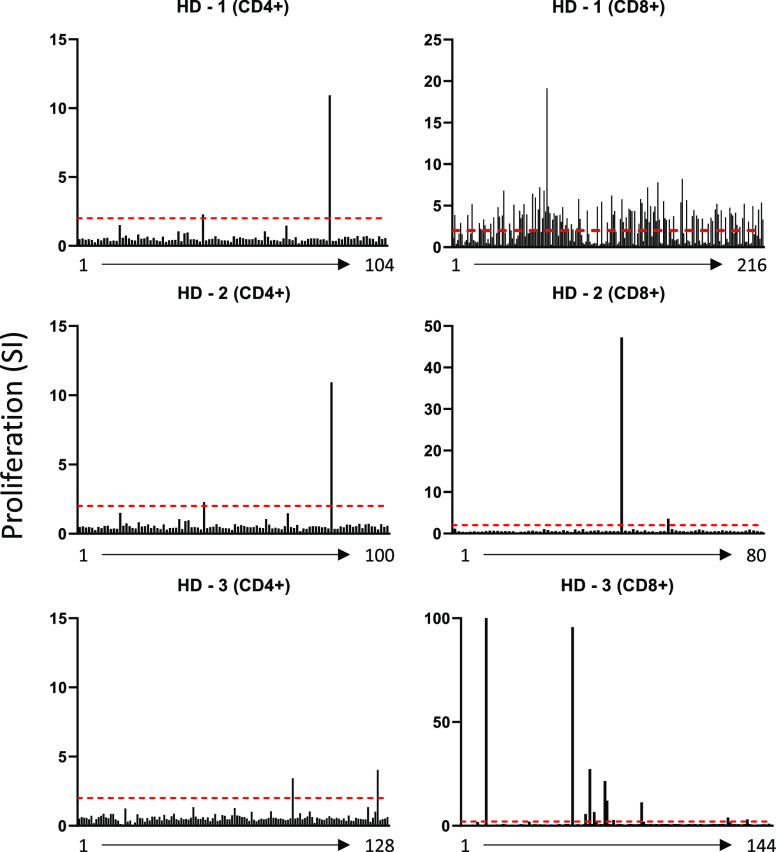
Proliferation of TCCs generated from HLA-A*32:01 positive donors following exposure to teicoplanin. T-cell populations were positively enriched for either CD4+ or CD8+ T-cells via magnetic bead separation (Miltenyi Biotec, UK). TCCs were rechallenged with 250 μM teicoplanin or cell culture medium for 48 h in the presence of autologous antigen presenting cells (Epstein–Barr virus-transformed B-cells; APCs). [^3^H]Thymidine was added for the final 16 h of incubation to measure proliferation, and clones with a stimulation index (SI) > 2 were deemed to be drug-responsive.

Following pretreatment of both APCs and T-cells with anti-HLA blocking antibodies, proliferation of CD8+ TCCs was unaffected after the HLA class II blockade (HLA-DP, HLA-DQ, and HLA-DR). However, proliferation was found to be inhibited in the presence of MHC class I blocking antibodies (Figure 2A) indicating T-cell responses to teicoplanin are driven primarily by MHC class I complexes. Autologous APCs pulsed with teicoplanin (30 min, 1 h, 4 h, and 24 h) displayed no proliferative response following coculture with teicoplanin-reactive TCCs (Figure 2B). After fixation of APCs with glutaraldehyde and subsequent attenuation of peptide processing pathways, drug-responsive T-cells exhibited the capacity for proliferation after exposure to a coculture of fixed APCs and teicoplanin. These data suggest teicoplanin is able to activate CD8+ TCCs in a processing independent manner in which direct pharmacological interactions with MHC, concordant with the p-i concept, evoke T-cell responses to drug.

**Figure 2:**
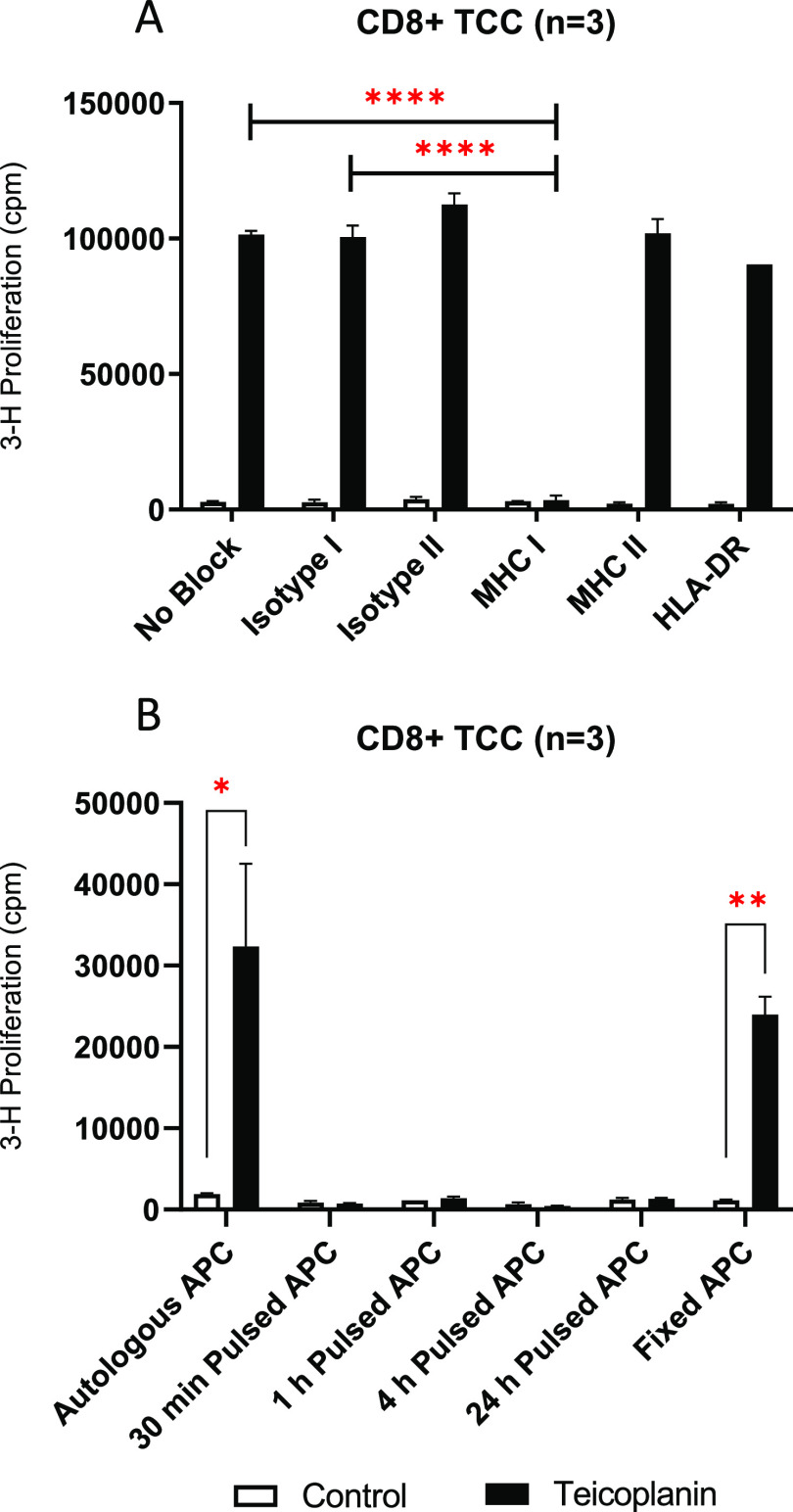
HLA restriction and activation pathway of teicoplanin-responsive CD8+ TCCs from HD-3. A) Proliferation in response to teicoplanin (250 μM) was measured following blocking of HLA complexes present on the surface of both APCs and TCCs using anti-HLA antibodies (BD Pharmingen, San Jose, USA) at a concentration of 10 μg/mL. B) Autologous APCs were either pulsed with teicoplanin for multiple time-points and extensively washed to remove unbound drug or fixed with glutaraldehyde to inhibit APC peptide processing. TCCs were then incubated for 48 h with pulsed APCs or fixed APCs plus teicoplanin (250 μM), with unmodified autologous APCs used as a positive control. [^3^H]Thymidine was added for the final 16 h of incubation to measure proliferative responses. Data is shown for representative TCCs (*n* = 3), and statistical significance was determined using the Mann–Whitney U test (*p < 0.05, **p < 0.01, ****p < 0.0001).

Cytokine and cytolytic molecule secretion of teicoplanin-reactive TCCs was assessed via ELISpot after a drug rechallenge (Figure 3A). Clones were observed to secrete both Th1 (IFN-γ) and Th2 (IL-5 and IL-13) cytokines. However, the secretion of Th17 and Th22 associated cytokines such as IL-17A and IL-22 was not present (data not shown). Interestingly, secretion of cytolytic molecules was detected in all TCCs profiled. Most notably, increased secretion of granzyme B, perforin, and FasL indicated involvement of cytotoxic T-cell responses and potential for activation of pro-apoptotic pathways. A cross-reactivity study of clones initially primed and exhibiting proliferative responses to teicoplanin revealed that memory T-cell responses to teicoplanin were associated with a greater degree of proliferation. Interestingly, TCCs exhibited cross-reactivity with the cyclic lipoglycopeptide, daptomycin, at graded concentrations. However, no cross-reactive T-cells were identified after exposure to vancomycin (Figure 3B).

**Figure 3:**
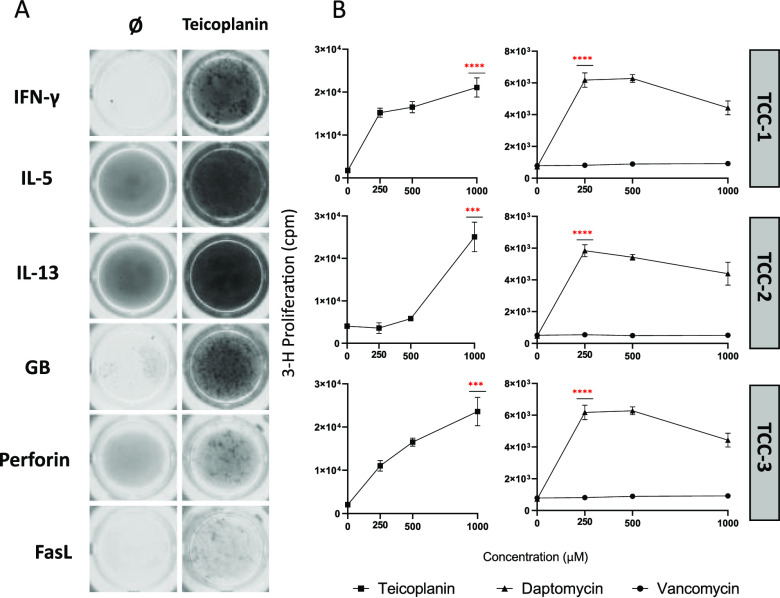
Cytokine/cytolytic molecule secretion profile and glycopeptide cross-reactivity of CD8+ teicoplanin-reactive TCCs from HD-3. A) Drug-responsive clones were incubated with autologous APCs and either teicoplanin (250 μM) or cell culture medium for 48 h (representative TCCs shown). T-cell secretion of cytokines (IFN-γ, IL-5, and IL-13) and cytolytic molecules (granzyme B, perforin, and FasL) was visualized via the enzyme-linked immunospot (ELISpot) assay using an ELISpot plate precoated for the cytokines of interest and developed according to the manufacturer’s instructions (Mabtech, Sweden). B) Cross-reactivity of teicoplanin-responsive T-cells to glycopeptides (vancomycin and daptomycin) was measured via the proliferation assay as previously described in Figures 1 and 2. Statistical significance was determined using a nonparametric *t*-test (***p < 0.001, ****p < 0.0001).

In summary, teicoplanin-responsive T-cells displaying a CD8+ phenotype were generated from 3 drug-naïve healthy donors expressing the HLA-A*32:01 allele, recently associated with cases of vancomycin-induced DRESS. Therapeutic concentrations associated with glycopeptide administration are typically between 10 and 20 μM, substantially lower than the optimal doses used within this study to elicit maximal T-cell responses for functional analysis. However, we have observed that glycopeptide-specific TCCs are capable of eliciting proliferative responses at lower, more therapeutically relevant doses in line with concentrations found within the blood plasma of patients. The identification of TCCs that proliferate and secrete both cytotoxic and DRESS related cytokines such as IL-5 suggests T-cell involvement within the pathogenesis of the teicoplanin-induced DRESS syndrome.^10^

Mechanistic T-cell assays revealed a processing independent mechanism of activation that hinges on drug presentation via direct interaction with HLA class I molecules. These data are concordant with previous mechanistic findings relating to T-cell responses to vancomycin for which it has been hypothesized glycopeptide compounds possess the capacity to displace and mimic native HLA peptides.^7^ Proliferative T-cell cross-reactivity of teicoplanin-responsive TCCs generated from healthy volunteers to daptomycin highlights the complex patterns of reactivity encountered within clinical settings. The observed *in vitro* T-cell cross-reactivity may be explained by structural similarities between both teicoplanin and daptomycin, specifically the presence of a hydrophobic lipid chain. Conversely, vancomycin’s structure comprises a heptapeptide chain that crucially contains a disaccharide, composed of vancosamine and glucose, instead of the lipid tail found on both teicoplanin and daptomycin molecules. This potentially explains why some teicoplanin-specific T-cells are able to proliferate in the presence of daptomycin but not vancomycin. One intriguing avenue to explore the nature of these cross-reactive responses involves the study of cellular energetic parameters, such as glycolysis, which may provide greater sensitivity for the determination of T-cell activation thresholds upon antigen presentation. However, to investigate the specificity of teicoplanin for HLA-A*32:01, additional cloning experiments focusing on individuals negative for HLA-A*32:01 expression will need to be conducted. Further genetic studies and functional T-cell analysis following HLA-glycopeptide binding will be required to determine the full pathway of glycopeptide cross-reactivity in addition to the extent of interactions with HLA-A*32:01 in order to predict potential susceptibility to severe cross-reactivity and improve patient safety.

## Abbreviations

ADR: adverse drug reaction
APCs: antigen presenting cells
DRESS: drug-reaction with eosinophilia and systemic symptoms
PBMCs: peripheral blood mononuclear cells
HLA: human leukocyte antigen
SI: stimulation index
TCC: T-cell clone

## References

[ref1] Pavlos R.; Mallal S.; Ostrov D.; Buus S.; Metushi I.; Peters B.; Phillips E. T cell-mediated hypersensitivity reactions to drugs. Annu. Rev. Med. 2015, 66, 439–454. 10.1146/annurev-med-050913-022745. 25386935 PMC4295772

[ref2] Campoli-Richards D. M.; Brogden R. N.; Faulds D. Teicoplanin. A review of its antibacterial activity, pharmacokinetic properties and therapeutic potential. Drugs 1990, 40, 449–486. 10.2165/00003495-199040030-00007. 2146108

[ref3] Wood M. J. The comparative efficacy and safety of teicoplanin and vancomycin. J. Antimicrob. Chemother. 1996, 37, 209–222. 10.1093/jac/37.2.209. 8707731

[ref4] Konvinse K. C.; Trubiano J. A.; Pavlos R.; James I.; Shaffer C. M.; Bejan C. A.; Schutte R. J.; Ostrov D. A.; Pilkinton M. A.; Rosenbach M.; Zwerner J. P.; Williams K. B.; Bourke J.; Martinez P.; Rwandamuriye F.; Chopra A.; Watson M.; Redwood A. J.; White K. D.; Mallal S. A.; Phillips E. J. HLA-A*32:01 is strongly associated with vancomycin-induced drug reaction with eosinophilia and systemic symptoms. Journal of allergy and clinical immunology 2019, 144, 183–192. 10.1016/j.jaci.2019.01.045. PMC6612297 30776417

[ref5] Kwon H. S.; Chang Y. S.; Jeong Y. Y.; Lee S. M.; Song W. J.; Kim H. B.; Kim Y. K.; Cho S. H.; Kim Y. Y.; Min K. U. A case of hypersensitivity syndrome to both vancomycin and teicoplanin. J. Korean Med. Sci. 2006, 21, 1108–1110. 10.3346/jkms.2006.21.6.1108. 17179696 PMC2721938

[ref6] Hsiao S. H.; Chen H. H.; Chou C. H.; Lin W. L.; Liu Yeh P. Y.; Wu T. J. Teicoplanin-induced hypersensitivity syndrome with a preceding vancomycin-induced neutropenia: a case report and literature review. J. Clin Pharm. Ther 2010, 35, 729–732. 10.1111/j.1365-2710.2009.01124.x. 21054466

[ref7] Ogese M. O.; Lister A.; Gardner J.; Meng X.; Alfirevic A.; Pirmohamed M.; Park B. K.; Naisbitt D. J. Deciphering adverse drug reactions: in vitro priming and characterization of vancomycin-specific T-cells from healthy donors expressing HLA-A*32:01. Toxicol. Sci. 2021, 183, 139 10.1093/toxsci/kfab084. 34175955 PMC8404995

[ref8] Nakkam N.; Gibson A.; Mouhtouris E.; Konvinse K.; Holmes N.; Chua K. Y.; Deshpande P.; Li D.; Ostrov D. A.; Trubiano J.; Phillips E. J. Cross-reactivity between vancomycin, teicoplanin and telavancin in HLA-A*32:01 positive vancomycin DRESS patients sharing an HLA-Class II haplotype. J. Allergy Clin. Immunol. 2021, 147, 403 10.1016/j.jaci.2020.04.056. 32439433 PMC7674263

[ref9] Mauri-Hellweg D.; Bettens F.; Mauri D.; Brander C.; Hunziker T.; Pichler W. J. Activation of drug-specific CD4+ and CD8+ T cells in individuals allergic to sulfonamides, phenytoin, and carbamazepine. J. Immunol. 1995, 155, 462–472. 7602118

[ref10] Choquet-Kastylevsky G.; Intrator L.; Chenal C.; Bocquet H.; Revuz J.; Roujeau J. C. Increased levels of interleukin 5 are associated with the generation of eosinophilia in drug-induced hypersensitivity syndrome. Br J. Dermatol 1998, 139, 1026–1032. 10.1046/j.1365-2133.1998.02559.x. 9990366

